# The relationship between non-alcoholic fatty liver and skeletal muscle mass to visceral fat area ratio in women with type 2 diabetes

**DOI:** 10.1186/s12902-019-0404-1

**Published:** 2019-07-17

**Authors:** Xiaoyou Su, Jing Xu, Chao Zheng

**Affiliations:** Diabetes Center and Department of Endocrinology, The Second Affiliated Hospital and Yuying Children’s Hospital of Wenzhou Medical University, Lucheng District Wenzhou, Wenzhou, Zhejiang Province People’s Republic of China

**Keywords:** Non-alcoholic fatty liver, Skeletal muscle mass, Visceral fat area, Skeletal muscle mass to visceral fat area ratio, Type 2 diabetes

## Abstract

**Background:**

Sarcopenic obesity, central obesity combined with decreased skeletal muscle mass, is identified to be associated with metabolic syndrome and cardiovascular diseases; however, its role in the occurrence of non-alcoholic fatty liver disease (NAFLD) among patients with type 2 diabetes mellitus (T2DM) remains unclear. Therefore, this study aimed to investigate the value of the skeletal-to-visceral ratio (SVR) in the prediction of NAFLD in T2DM.

**Methods:**

T2DM patients (*n* = 445) were recruited into the current study. Hepatic steatosis was diagnosed based on ultrasonic results, while skeletal muscle mass as well as visceral fat area (VFA) was estimated based on bioimpedance analysis measurements.

**Results:**

NAFLD prevalence increased with the decreased SVR tertiles: statistically significant differences were observed in the highest tertiles (21.5% in men, and 30.4% in women) and the lowest tertiles (53.9% in men and 60.0% in women) (both *P* < 0.01). The decreased SVR tertiles were independently associated with the presence of NAFLD in female T2DM patients, with the odds ratio (OR) of 3.43 and 2.31 in the lowest and middle tertiles, respectively. Besides, the areas under the curve (AUC) for identifying NAFLD were 0.675 and 0.63 in men and women, respectively (*P* < 0.05).

**Conclusions:**

T2DM patients who have lower SVR levels are associated with higher risks of developing the NAFLD-related complications. Besides, SVR shows independent correlation with NAFLD in female T2DM patients, suggesting that SVR may be a useful index to predict the high risk of hepatic steatosis in T2DM.

## Background

Non-alcoholic fatty liver disease (NAFLD) is a disorder characterized by excess hepatic accumulation of fat in subjects without alcohol abuse history (i.e., < 20 g alcohol is consumed daily) [1]. Recently, NAFLD becomes prevalent across the world, whereas the underlying mechanisms have not been completely comprehended yet. NAFLD morbidity among various races is estimated to be about 15–30% [2–4]. Nevertheless, the prevalence of NAFLD will rapidly increase in the case of concurrent type 2 diabetes mellitus (T2DM). According to report, over 70% T2DM patients will develop NAFLD at the same time [5]. China is encountered with a severe problem. As shown in one study, the prevalence of NAFLD combined with T2DM is almost 80% among the Chinese [6]. Diabetes shows an independent association with the genesis as well as the development of NAFLD [7]. In addition, NAFLD exerts certain harmful influence on T2DM, such as poorer glucolipid metabolism, and increase risks of macro- or micro-vascular complications [8]. As a result, it is important to identify individuals at risk for NAFLD and institute timely intervention to prevent disease progression, particularly in the case of combined T2DM.

In an aging population, sarcopenia will lead to higher possibilities of developing metabolic disease as well as premature mortality [9, 10]. Additionally, accumulation of visceral fat has been identified to lead to a higher probability of developing NAFLD [10]. In recent years, sarcopenia has been identified to be accompanied by visceral obesity, also known as sarcopenic obesity. Sarcopenic obesity will present a dual metabolic burden, which has emerged as a major concern for public health [11]. In some existing studies, NAFLD was suggested to be related to sarcopenia obesity among the general population [12]. However, the connection of sarcopenia obesity with NAFLD remains unclear among T2DM patients.

Given the close correlation of sarcopenic obesity with cardiovascular disease (CVD) [13], it may be a useful index to estimate abdominal fat as well as skeletal muscle by dividing the appendicular skeletal muscle (ASM) by the visceral fat area (VFA), known as the skeletal-to-visceral ratio (SVR) to simply indicate sarcopenic obesity [14]. Therefore, the current work aimed to explore the connection of SVR with hepatic steatosis among T2DM patients.

## Methods

### Patients

The subjects in this cross-sectional study were 445 Chinese patients with T2DM (40 years≤ age ≤ 75 years). We consecutively recruited the subjects who visited the Second Hospital Affiliated to Wenzhou Medical University for evaluation or treatment of T2DM from April 2017 to September 2017. Patients receiving dual bioelectrical impedance analysis were screened, and then they underwent abdominal ultrasonography (IU22, Philips, healthcare, Andover and MA) using the 3.5 MHz transducer after fasting for 8 h. In addition, patients suffering from kidney dysfunction (with the estimated glomerular filtration rate, GFR, of < 30 ml/min/1.73 m2), nutritional compromises (like cancer, thyroid disease, skeletal deformity or amputation), alcohol abuse (> 140 g/week) as well as history of chronic viral hepatitis B (CHB) or C (CHC) infection confirmed by serologic markers were excluded from the current study. We have received the agreement from all subjects that they were willing to participate in this study and the informed consent was provided too. This study has gained approval from the Institutional Review Board of the Second Affiliated Hospital of Wenzhou Medical University (No. LCKY2017–01).

### Anthropometric and biochemical measurements

Physical examination consisted of anthropometric measurements (including body height, weight, and waist), as well as blood pressure (BP) assessment. Height was measured to the nearest 0.1 cm with a freestanding wood stadiometer. Weight was measured to the nearest 100 g with mechanical scales. Waist circumference was measured as the smallest circumference between the ribs and the iliac crest while the patient was standing with the abdomen relaxed, at the end of a normal expiration. In addition, the body mass index (BMI) would be measured as the ratio of weight (kg) to height squared (m2). BP (mmHg) would be detected by a mercury sphygmomanometer after resting for 5 min in the supine position. In addition, the levels of glycosylated hemoglobin (HbA1c), total cholesterol (TC), triglyceride (TG), high density lipoprotein cholesterol (HDL-C), low density lipoprotein cholesterol (LDL-C), alanine aminotransferase (ALT), as well as aspartate aminotransferase (AST), were also calculated using auto analyzer after fasting for > 8 h.

### Measurements of muscle mass and fat mass with a dual bioelectrical impedance analyzer

The lean body mass of the arms and legs, ASM, as well as VFA were calculated using a dual bioelectrical impedance analyzer (BIA) (InBody 720; Biospace, land Seoul, Korea). The analyzer measured resistance at specific frequencies (1, 5, 50, 250, 500 KHz, and 1 MHz) and reactance at specific frequencies (5,50, and 250KHz). BIA is now considered an accurate method for body composition evaluation [15]. In accordance with previous researches, there existed good connection between VFA measured by a BIA and that measured by an abdominal computed tomography (CT) [16]. ASM (kg) could be defined as the sum of the lean soft tissue masses of the arms and legs on the basis of Heymsfield et al. [17]. SVR (g/cm2) was calculated as an index of sarcopenic obesity by classifying the ASM (g) by VFA (cm2).

### Statistical analysis

The SPSS statistical software (Version 21.0, SPSS, Inc., Chicago, IL, USA) had been utilized for statistical analysis. The absolute volume of skeletal muscle as well as VFA was different between genders; as a results, men and women were independently analyzed. Besides, numerical variables would be presented in the form of mean ± SD or median [inter-quartile range], whereas categorical variables would be expressed as percentage. One-way ANOVA analysis was employed to compare the means, while Pearson’s chi-squared test would be adopted to compare the proportions. In addition, the connection of SVR level with the rest parameters would be determined by Spearman correlation analysis. Afterwards, the multivariate logistic regression models would be established in accordance with the SVR tertiles (namely ≤183, 183–237 and ≥ 237 Kg/cm2 in men; as well as ≤124, 124–155, and ≥ 155 Kg/cm2 in women), so as to assess the odds ratio (OR) regarding NAFLD. Specifically, the potential confounders had been in the above-mentioned models, including age, course of diabetes, BMI, waist, SBP, DBP, HbA1c, smoking, alcohol consumption, ALT, AST, TC, TG, HDL-C and LDL-C. Typically, different with a *p*-value of < 0.05 had been considered to be of statistical significance.

## Results

### Characteristics of participants

The clinical as well as metabolic characteristics of patients are listed in Table 1. 41.4% out of the 445 T2DM patients receiving abdominal ultrasound had been diagnosed with undergoing NAFLD. Patients in NAFLD group were associated with a remarkably higher weight, BMI, waist, ALT, AST, VFA and ASM compared with those without NAFLD. Otherwise, SVR would be evidently decreased in NAFLD patients.

**Table 1:** Baseline characteristics of type 2 diabetic patients with or without NAFLD

	Men			Women		
	NAFLD(−)	NAFLD(+)	P	NAFLD(−)	NAFLD(+)	P
N	147	89	–	113	96	–
Age, years	57.6 ± 9.4	59.4 ± 9.8	0.169	61.1 ± 9.3	61.4 ± 8.7	0.834
Duration of diabetes, years	8.0 ± 7.1	6.1 ± 6.1	0.040	9.1 ± 6.6	7.7 ± 6.7	0.143
Duration of hypertension, years	3.1 ± 5.6	5.5 ± 7.1	0.05	5.0 ± 6.7	6.9 ± 7.0	0.045
Height, cm	167.9 ± 5.7	167.5 ± 5.1	0.596	154.9 ± 5.7	155.9 ± 5.7	0.201
Weight, cm	65.0 ± 9.1	73.6 ± 10.2	< 0.001	58.3 ± 9.1	63.3 ± 11.7	0.001
Body mass index, Kg/m^2^	23.0 ± 2.9	26.2 ± 3.4	< 0.01	24.3 ± 3.5	26.0 ± 4.4	0.002
Waist circumference, cm	85.6 ± 8.5	94.7 ± 8.4	< 0.001	86.8 ± 11.8	92.2 ± 11.4	0.001
Systolic blood pressure, mmHg	133.2 ± 21.8	138.4 ± 20.6	0.069	145.7 ± 26.4	140.8 ± 19.3	0.133
Diastolic blood pressure, mmHg	76.0 ± 12.5	80.7 ± 12.4	0.005	75.7 ± 12.8	76.8 ± 12.6	0.529
Hemoglobin A1c, mmol/L	10.3 ± 2.8	9.6 ± 2.2	0.005	9.4 ± 2.0	9.4 ± 2.1	0.901
Total cholesterol, mmol/L	4.2 ± 1.1	4.6 ± 1.3	0.016	4.6 ± 1.4	4.9 ± 1.4	0.050
Triglycerides, mmol/L	1.4 ± 0.9	2.3 ± 2.2	< 0.001	1.9 ± 2.1	2.3 ± 1.8	0.155
HDL-cholesterol, mmol/L	1.0 ± 0.3	0.9 ± 0.2	0.152	1.1 ± 0.3	1.0 ± 0.2	0.390
LDL-cholesterol, mmol/L	2.5 ± 0.9	2.6 ± 1.0	0.201	2.6 ± 0.9	2.8 ± 0.9	0.061
ALT, IU/L	24.2 ± 14.5	28.5 ± 18.2	0.047	20.8 ± 11.8	25.0 ± 14.0	0.039
AST, IU/L	22.6 ± 10.5	25.4 ± 11.5	0.054	22.1 ± 9.7	25.0 ± 11.2	0.049
Current smoking, %	44.2	43.8	1.000	1.8	0	0.501
Current drinking, %	10.2	11.2	0.829	0	0	1.000
Menopause, %	–	–	–	89.2	91.3	0.645
Medications						
Statin (%)	8.2	11.1	0.717	10.4	8.3	1.000
Metformin (%)	53.1	61.1	0.512	50.0	61.1	0.378
Insulin (%)	65.3	50.0	0.185	70.8	63.9	0.637
ASM, kg	21.5 ± 2.8	22.6 ± 2.7	0.002	15.4 ± 2.4	15.7 ± 2.6	0.369
Visceral fat area, cm^2^	95.7 ± 25.6	120.7 ± 36.0	< 0.001	104.7 ± 32.9	121.0 ± 28.8	< 0.001
SVR, g/cm^2^	238.7 ± 73.3	197.8 ± 57.4	< 0.001	160.0 ± 58.0	136.7 ± 38.1	0.001

### Pearson correlation between different variables and SVR

Correlation of SVR with the clinical as well as biochemical metabolic variables can be found in Table 2. As could be observed, SVR was negatively associated with age, BMI, and waist circumference regardless of gender. Otherwise, among male patients, SVR displayed markedly negative correlation with SBP.

**Table 2:** Correlation analysis between SVR levels and other variables

	Men		women	
	r	*P* value	r	*P* value
Age	−0.377	< 0.001	−0.332	< 0.001
Body mass index	−0.244	< 0.001	−0.270	< 0.001
Waist circumference	−0.309	< 0.001	−0.328	< 0.001
SBP	−0.179	< 0.001	−0.053	0.255
DBP	− 0.057	0.195	− 0.070	0.136
Glycosylated hemoglobin A1c	0.073	0.103	−0.033	0.491
Total cholesterol	−0.022	0.613	−0.089	0.059
Triglycerides	0.062	0.159	−0.038	0.412
HDL-cholesterol	0.019	0.663	−0.025	0.591
LDL-cholesterol	−0.028	0.519	−0.059	0.207

### Comparison of the incidence of NAFLD in different tertiles of SVR

**Fig. 1:**
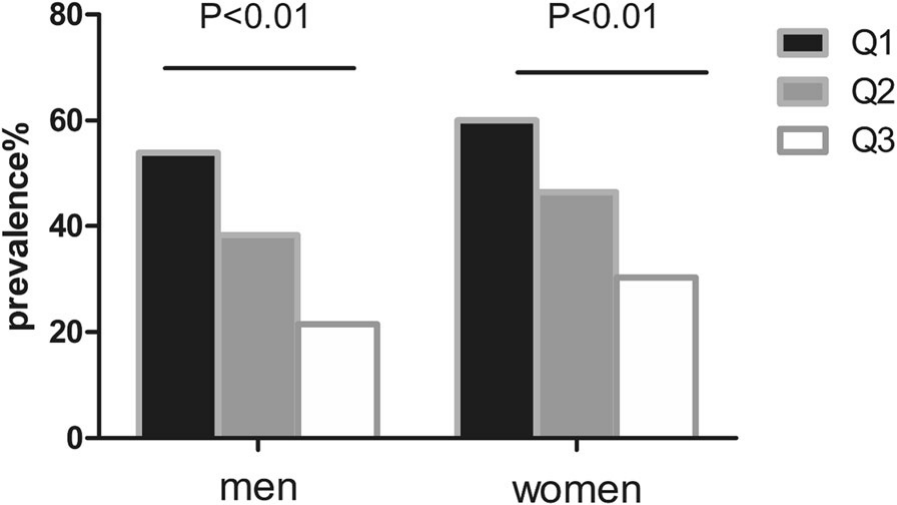
prevalence of NAFLD across tertiles of SVR in Chinese type 2 diabetic patients

The prevalence of NAFLD tended to decline with the increase in SVR (Fig. 1). Women were associated with a higher incidence of NAFLD than men. For male patients, the prevalence of NAFLD at Q1, Q2 and Q3 levels would be 53.9, 38.3 and 21.5%, respectively, while that for female patients would be 60.0, 46.4 and 30.4%, respectively.

### Logistic regression analyses for NAFLD

The results of logistic regression analysis for determining the relationship of SVR tertiles with NAFLD are listed in Table 3. As for the non-adjusted model, the OR for NAFLD would be 4.27 (95% CI, 2.12–8.61) in men and 3.43 in women (95%CI, 1.70–6.91) with regard to the smallest versus the largest SVR tertile. The ORs had been reduced after adjustment for age, duration of diabetes, BMI, waist circumference, SBP, DBP, HbA1c, smoking status, alcohol intake, ALT, AST, TC, TG, HDL-C, as well as LDL-C; however, the relationship of SVR with NAFLD was still obvious in female patients (OR = 3.43, *P* = 0.020), but, that among male patients was not significant.

**Table 3:** ORs (95%CI) for NAFLD in type 2 diabetes according to SVR tertiles

	Univariate		Model1		Model2	
	OR	P	OR	P	OR	P
Men						
Q1	4.27(2.12–8.61)	0.000	4.79(2.17–10.56)	0.000	2.83(0.55–8.43)	0.213
Q2	2.26(1.12–4.55)	0.022	2.38(1.16–4.88)	0.018	1.43(0.40–5.17)	0.581
Q3	1(reference)	–	1	–	1	–
women						
Q1	3.43(1.70–6.91)	0.001	4.41(1.99–9.74)	0.001	3.43(1.41–8.74)	0.020
Q2	1.98(0.98–3.97)	0.056	2.41(1.13–5.13)	0.023	2.31(1.01–5.63)	0.046
Q3	1	–	1	–	1	–

Model1: adjusted for age

Model2: further adjusted for duration diabetes, BMI, waist, SBP, DBP, HbA1c, smoker, alcohol, ALT, AST, TC, TG, HDL-C, LDL-C, and medication history for diabetes and dyslipidemia

### ROC curves of SVR for NAFLD

**Fig. 2:**
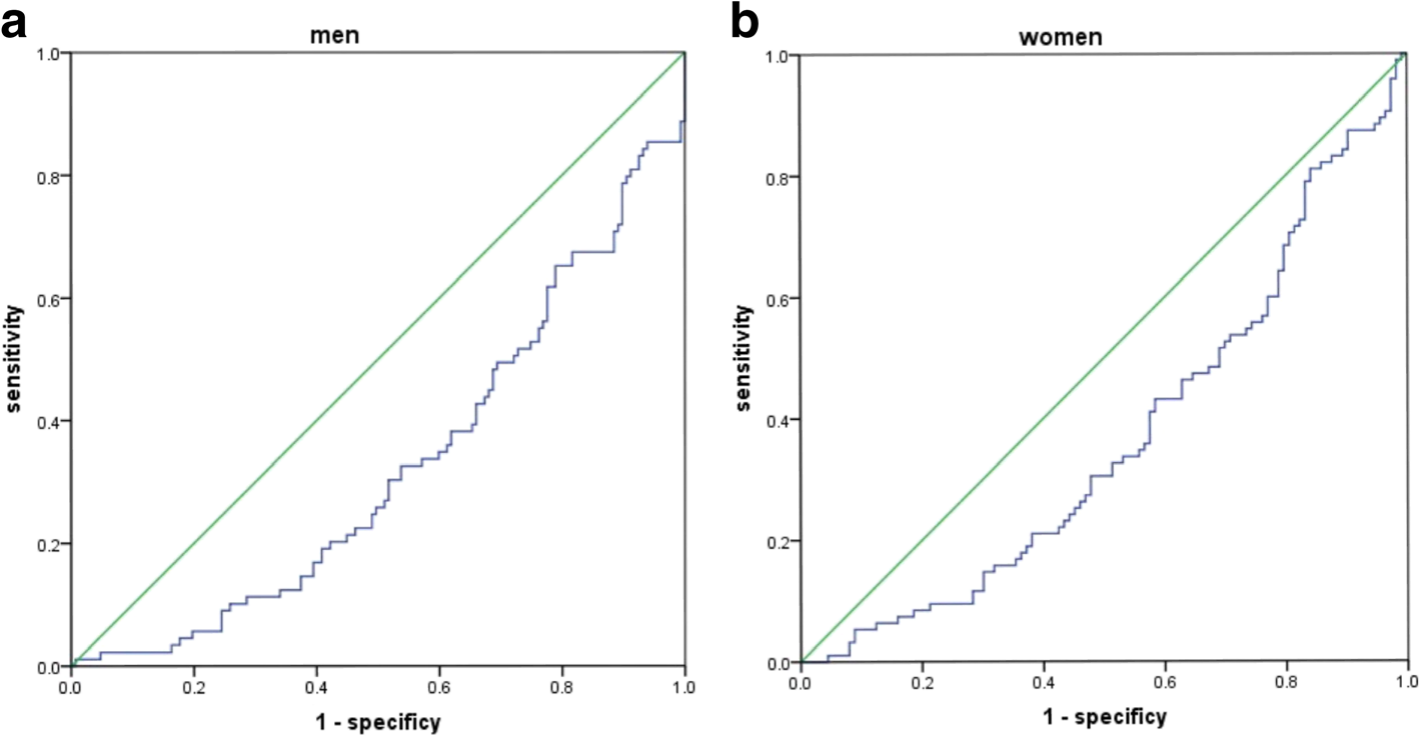
**a** ROC analysis of SVR to NAFLD among men patients. **b** ROC analysis of SVR to NAFLD among women patients

The role of SVR in diagnosing NAFLD had been determined through the ROC curve (Fig. 2a-b), and the AUC was 0.675 for men while 0.632 for women (*P* < 0.01). The cutoff with the biggest Youden index of SVR was 203Kg/cm2, with the sensitivity of 61.8% and the specificity 65.3% in men. Besides, the cutoff of SVR was 139 Kg/cm2, with the sensitivity of 66.3% and the specificity 55.8% in women. The SVR threshold of ≤129 Kg/cm2 was associated with the sensitivity of 95% in men, while that of ≤92 Kg/cm2 had the sensitivity of 95% in women.

## Discussion

To the best of our knowledge, this is the first study to examine the correlation of SVR with NAFLD in Chinese T2DM patients. According to our results, SVR showed an independent association with NAFLD in female T2DM patients, which was still obvious following adjustment for SVR-associated factors related to NAFLD. Therefore, SVR could serve as a strong predictor for NAFLD.

T2DM will frequently lead to excessive body fat, along with enhanced loss of muscle mass [18, 19], which is termed as the sarcopenic obesity. Besides. NAFLD in T2DM individuals may be synergistically affected by the effect of high fat mass combined with low muscle mass. In recent years, Choe et al. suggested in their cross-sectional study that, elevated body fat was associated with the reduced muscle mass, as verified through the waist-to-calf circumference index, in the presence of elevated incidence of NAFLD among T2DM patients [20]. Simple correlation between the two has been indicated in prior studies, but muscle mass as well as VFA would be evaluated through the dual bioelectrical impedance analyzer in this study, which had been identified to exhibit higher accuracy in quantifying the muscle as well as fat mass [21]. In this study, the Q1 of SV ratio indicated the resembling of sarcopenic obesity; moreover, the issue of reduced skeletal muscle concurrent with increased visceral fat would also be discussed.

Obesity is remains a leading risk factor of NAFLD, and it is characteristic of ectopic hepatic fat accumulation. Our results suggested that, compared with diabetics with no NAFLD, those with NAFLD had higher weight, BMI, waist circumference and VFA. Moreover, Takashi Shida et al. demonstrated that, a low SVR level might be correlated with BMI, HOMA-IR, as well as cirrhosis among NAFLD patients [22]. Our study had discovered that, the SVR level had displayed inverse correlation with BMI as well as waist circumference among T2DM patients, which suggested that SVR might significantly affect the genesis as well as progression of T2DM patients with NAFLD.

As suggested in this study, a lower SVR level was linked with a higher prevalence of NAFLD than that in the higher SVR group, regardless of gender. Moreover, SVR would not be related to hepatic steatosis in men. Newman et al. argued that women were associated with a lower muscle mass while higher visceral fat than men, so they are more likely to develop functional limitations as well as disability related to sarcopenic obesity (including NAFLD), which is consistent with our finding [10, 23]. Otherwise, 90.1% of our female cases were postmenopausal with muscle mass loss or reduction, and were thereby at a higher risk of NAFLD [24].

Our findings on the connection of SVR with NAFLD should be further examined about their applicability among other ethnic groups, since the difference specific to ethnicity in the distribution between visceral and subcutaneous adipose tissues is detected in individuals with different races [25]. In the west, individuals undergoing incident sarcopenic obesity only take up 5–10% [26], As a result, more research is needed to examine the association of SVR with NAFLD development among the non-Chinese populations.

Some reasonable mechanisms may be responsible for the association of SVR, the sarcopenic obesity indicator, with NAFLD. Initially, skeletal muscle accounts for the primary place of glucose disposal mediated by insulin, so the reduced muscle mass may be correlated with insulin resistance [27]. Additionally, visceral fat accumulation has been suggested to be related to insulin resistance [28]. As a consequence, a low SVR level may display connection to insulin resistance. Secondly, muscle mass reduction, together with visceral fat accumulation, is correlated with the reduced physical activities [29]. Therefore, insulin resistance and physical inactivity, may account for the pathophysiology regarding the marked correlation of SVR with NAFLD. Our results of logistic regression analysis based on various parameters demonstrated that the OR for NAFLD was 3.43 folds greater in low SVR tertile compared with that in high SVR tertile among females.

However, this study still has some shortcomings. Firstly, this study is a cross-sectional study, so the causality should not be determined. Secondly, the dual bioelectrical impedance analyzer was employed to measure VFA. In addition, abdominal CT scan has been regarded as the gold standard to assess visceral adiposity. In a previous study, bioelectrical impedance analysis may underestimate VFA in obese subjects [30]. Thirdly, this study did not examine the impact of dietary intake and physical activity. Fourthly, the small sample size in this study was insufficient to make definite conclusions.

## Conclusions

In conclusions, our results reveal that, a lower SVR level is independently associated with the increased risk of NAFLD among female T2DM patients. Moreover, SVR can be a factor predicting NAFLD, which may be used to help doctors to monitor NAFLD development in clinical practice.

## Data Availability

The data that support the findings of this study are available from Institutional Review Board of the second affiliated hospital and Yuying Children’s Hospital of Wenzhou Medical University but restrictions apply to the availability of these data, which were used under license for the current study, and so are not publicly available. Data are however available from the authors upon reasonable request and with permission of Institutional Review Board of the Second Affiliated Hospital and Yuying Children’s Hospital of Wenzhou Medical University.

## Abbreviations

ALT: Alanine aminotransferase
ASM: Appendicular skeletal muscle
AST: Aspartate aminotransferase
HbA1c: Glycosylated hemoglobin
HDL-C: High-density lipoprotein cholesterol
LDL-C: Low-density lipoprotein cholesterol
NAFLD: Non-alcoholic fatty liver disease
SVR: Skeletal muscle mass to visceral fat area ratio
TC: Total cholesterol (TC)
TG: Triglyceride
VFA: Visceral fat area
